# The independence of eye movements in a stomatopod crustacean is task dependent

**DOI:** 10.1242/jeb.153692

**Published:** 2017-04-01

**Authors:** Ilse M. Daly, Martin J. How, Julian C. Partridge, Nicholas W. Roberts

**Affiliations:** 1 School of Biological Sciences, University of Bristol, Tyndall Avenue, Bristol BS8 1TQ, UK; 2 School of Animal Biology and the Oceans Institute, Faculty of Science, University of Western Australia, 35 Stirling Highway (M317), Crawley, WA 6009, Australia

**Keywords:** Mantis shrimp, Visual system, Gaze stabilization, Optokinesis, Saccade, Independent eyes, Neural connections

## Abstract

Stomatopods have an extraordinary visual system, incorporating independent movement of their eyes in all three degrees of rotational freedom. In this work, we demonstrate that in the peacock mantis shrimp, *Odontodactylus scyllarus*, the level of ocular independence is task dependent. During gaze stabilization in the context of optokinesis, there is weak but significant correlation between the left and right eyes in the yaw degree of rotational freedom, but not in pitch and torsion. When one eye is completely occluded, the uncovered eye does not drive the covered eye during gaze stabilization. However, occluding one eye does significantly affect the uncovered eye, lowering its gaze stabilization performance. There is a lateral asymmetry, with the magnitude of the effect depending on the eye (left or right) combined with the direction of motion of the visual field. In contrast, during a startle saccade, the uncovered eye does drive a covered eye. Such disparate levels of independence between the two eyes suggest that responses to individual visual tasks are likely to follow different neural pathways.

## Abstract (precis)

**Summary:** The level of independence between the eyes of mantis shrimps (stomatopods) is task dependent, suggesting variability in neural processing of visual information.

## INTRODUCTION

It is not unusual for animals to have eyes that move independently of one another. Chameleons are perhaps the most famous example (Tauber and Atkin, 1967), but there are also examples of ocular independence in teleost fish (Land, 1999a; Pettigrew et al., 1999; Fritsches and Marshall, 2002), reptiles (Pettigrew et al., 1999) and crustaceans (Land et al., 1990; Cronin and Marshall, 2001), to name but a few. The degree of independence between an animal's eyes can vary depending on the task it is performing. For instance, a chameleon's eyes will behave independently whilst surveying its general surroundings, but during tracking or ocular pursuit of a target, the two eyes can become yoked together to display conjugate eye movements (Katz et al., 2015). Similarly, the eyes of the pipefish *Corythoichthyes intestinalis* show disconjugate movement during scene surveying, yet the two eyes move conjugately during gaze stabilization (Fritsches and Marshall, 2002). In contrast, the sandlance *Limnichthyes fasciatus* has eyes that are apparently completely independent, even during gaze stabilization (Fritsches and Marshall, 2002).

**Fig. 1.**
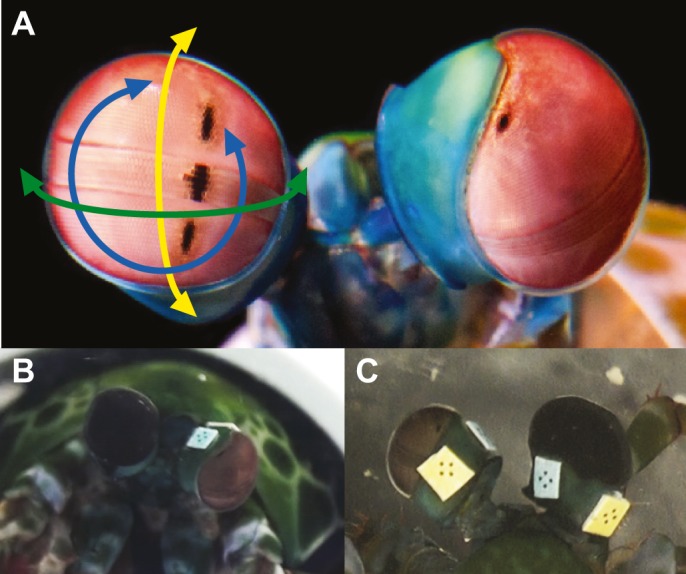
**A frontal view of the colourful eyes of *Odontodactylus scyllarus*.** (A) Each eye is capable of >90 deg independent rotation in the yaw (green arrow), pitch (yellow arrow) and torsion (blue arrow) directions. Image courtesy of Mike Bok. (B,C) A view of an experimental animal from (B) the front and (C) above during the covered phase of the experiment, in which one eye was completely occluded with black nail varnish. This animal had its right eye occluded.

Stomatopods too show independent movement of their left and right eyes (Cronin et al., 1988; Land et al., 1990; Jones, 1994). Their visual system is extraordinarily complex, with each apposition compound eye divided into three sections: the dorsal and ventral hemispheres, and a two to six ommatidial row midband (depending on the species) bisecting the eye about its equator (Exner, 1891; Schiff, 1963; Horridge, 1978; Manning et al., 1984; Marshall, 1988; Marshall et al., 1991a,b, 2007; Chiou et al., 2008; Roberts et al., 2009; Thoen et al., 2014). Particular to each of these sections are regional specializations conferring both linear and circular polarization vision, as well as up to 12-channel colour vision (Marshall, 1988; Marshall et al., 1991a,b, 2007; Chiou et al., 2008; Roberts et al., 2009; How et al., 2014; Thoen et al., 2014). The axes of both eyes are capable of >90 deg rotation in each of the three degrees of rational freedom: yaw (side-to-side), pitch (up–down) and torsion (rotation about the visual axis) (Cronin et al., 1988; Land et al., 1990; Jones, 1994) (Fig. 1A). Their large repertoire of eye movements include gaze stabilization (optokinesis) and startle saccades in response to salient stimuli with both luminance and polarization contrast as well as short-duration scans to obtain spectral and polarization information (Land et al., 1990; Cronin et al., 1991; Marshall et al., 2014; Daly et al., 2016).

In this work, we investigated the extent to which the two eyes of the peacock mantis shrimp, *Odontodactylus scyllarus*, are independent in each degree of rotational freedom, and whether the level of independence depends on the type of visual task.

## MATERIALS AND METHODS

Individuals of the tropical stomatopod species *O. scyllarus* (Linnaeus 1758) (Crustacea: Stomatopoda) were presented with two types of high-contrast visual stimuli: (1) a wide-field black and white (square wave profile) moving grating and (2) a black-on-white looming stimulus. Each experiment consisted of two phases: ‘before occlusion’ (both eyes uncovered) followed by ‘after occlusion’ (one eye covered, one uncovered), in which one eye of an individual was completely occluded using opaque black nail varnish (800 Black Out, 60 Seconds Super Shine, Rimmel, London, UK; Fig. 1B,C). The same six animals were presented with both types of stimuli in order to directly compare within an individual the effect of occlusion on different visual tasks. Half of the animals were shown the moving grating stimulus prior to the looming stimulus, and half were shown the stimuli in the other order, with a 10 min rest period between each.

### Optokinesis

**Fig. 2.**
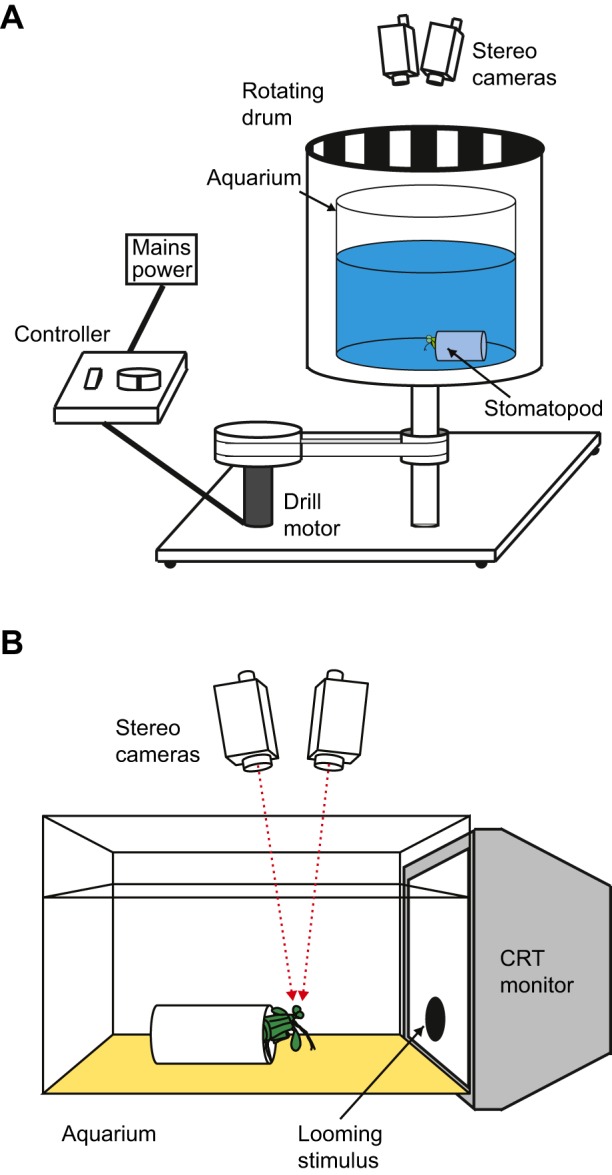
**Experimental set-up.** (A) The set-up for the optokinesis experiments, displaying a cutaway of the rotating drum to show the experimental aquarium and location of the stomatopod. The inner face of the drum was covered with a high-contrast grating, providing the animal with a horizontal field of motion when the drum was in motion. The movement of the eyes was recorded with a calibrated stereoscopic camera pair and tracked using the method described by Daly et al. (2016). (B) The experimental set-up for the looming experiment (modified from Daly et al., 2016), which involved presentations of a black circle that rapidly appeared on a CRT monitor. Eye tracking as for A.

Six *O. scyllarus* were individually placed within an artificial burrow with their heads at the centre of a 20 cm radius cylindrical aquarium fixed in the centre of a larger rotating drum (diameter 30 cm, height 40 cm), both constructed from transparent acrylic plastic (Fig. 2A). The aquarium was filled to a depth of 15 cm with seawater from the animal's home aquarium. The rotating drum was not filled with water and was free to spin about a vertical axis in either the clockwise or anticlockwise direction, driven by a drill motor (970D, Como Drills, Kent, UK) at an angular speed of 11.26±0.42 deg s^−1^ (mean±s.d.). The visual stimulus was provided by a square wave grating comprising 24 pairs of black and white stripes of equal width (1.96 cm) printed on A3 paper and attached to the inner side of the drum, with each stripe pair subtending a visual angle of 15 deg from the position of the experimental animal. The Michelson contrast of the stripes was 93.8% in the 420–700 nm range of the spectrum.

Each animal was presented with a moving drum six times: three with clockwise rotation of the drum and three with anticlockwise rotation. The three-dimensional movements of the eyes were recorded using a pair of calibrated stereoscopic video camcorders (Panasonic HC-X900, Osaka, Japan) and eye movements were tracked in the video recordings using MATLAB version 2015b (The MathWorks, Natick, MA, USA) and the method previously described by Daly et al. (2016).

The rotation of the drum causes wide-field motion of the visual field, which elicits optokinesis, a stereotypical gaze stabilization response from the stomatopod (Cronin et al., 1991). Optokinesis consists of ocular nystagmus movements, which comprise two phases: a slow phase in which the eye tracks the movement of the black and white grating, and a fast phase in which the eye performs a rapid counter-rotation to ‘reset’ its position. Gaze is stabilized during the slow phase, allowing the animal to perform a variety of visual tasks, whereas, because of the speed of the counter-rotation (>40 deg s^−1^), vision is thought to be suppressed during fast counter-rotation (Land et al., 1990). Gaze stabilization performance can be quantified using the relative velocity ratio (previously termed ‘gain’ by Cronin et al., 1991), which is the ratio between the velocity of the drum and the velocity of the eye. As the rotating drum is only free to rotate about the (vertical) *z*-axis, the angular velocity of the grating on the drum will be purely in the *x*–*y* (yaw) plane. Similarly, the angular velocity of the eye will be the velocity of rotation in the yaw (side-to-side) degree of freedom. The relative velocity ratio in the yaw plane, *S*_y_, is defined as the ratio of eye and drum angular velocities in this plane:
(1)
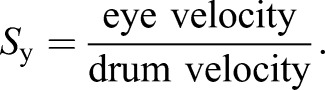
If the angular velocity (in deg s^−1^) of the eye in the yaw degree of freedom matches that of the drum, then *S*_y_=1, which corresponds to perfect gaze stabilization in which, from the perspective of the eye, the drum no longer appears to move. In reality, for the closed-loop system of response to be stable, the relative velocity ratio must be less than 1 (*S*_y_<1), because of the delay caused by the finite response time of the animal's visual system. *S*_y_>1 when the eye rotates faster than the drum, and if the eye is stationary then *S*_y_=0. If the eye rotates in the opposite direction to the drum, as it does during the fast reset phase of optokinetic nystagmus, then *S*_y_<0. The relative velocity ratio is a measure of the performance of an eye during gaze stabilization, and as such its calculation is, strictly speaking, applicable only to the eye movements during the slow phase of optokinesis, when the eye rotates in the same direction as the drum. Consequently, in the following statistical analyses, only values of *S*_y_ calculated during the slow phase of optokinesis are considered. However, values of *S*_y_ during both slow and fast phases are included in several figures to demonstrate the overall trend in yaw rotation of the eye during optokinesis.

### Looming

The same six *O. scyllarus* were presented with an unpolarized, luminance-only looming stimulus presented on a computer monitor, programmed in MATLAB (The MathWorks) using functions from the Psychophysics Toolbox library (Pelli, 1997). This consisted of a black circle that suddenly appeared and rapidly expanded (54.55 cm s^−1^ or 209 deg s^−1^) to a circle of diameter 12 cm (46 deg visual angle) on the white background of a cathode ray tube (CRT) monitor (S96D, Videoseven, Ingram, Machrotron Gmbh, Dornach, Germany). A CRT monitor was used to avoid the stimulus having any polarization contrast. The average Weber contrast in the 420–720 nm region of the spectrum was 98.6%. The animals were placed in artificial burrows ca. 14 cm from the CRT monitor in an aquarium filled with seawater from their home aquarium. The centre of the circle was at the same elevation as the eyes and coincided with the animal's midline (Fig. 2B).

Each animal was presented with six trials, one of which was a control in which the intensity of the looming circle matched the white background (thus presenting no visual stimulus). The time between the presentations of stimuli was varied randomly between 90 and 120 s. As for the optokinesis experiment, the three-dimensional rotation of the eyes was determined from the output of a calibrated pair of stereoscopic video cameras using the method previously described by Daly et al. (2016).

### Occlusion

Once each animal had been presented with both the grating and the looming stimuli, the same experimental procedures were repeated after occlusion of an eye, with a 30–40 min rest period between occlusion and the start of the stimulus. The nail varnish was applied to an eye using a size 00 paintbrush (0.5 mm, AG4030, Coloro Brush, Humbrol, Sandwich, Kent, UK). For experimental balance, the left eye of half of the experimental animals and the right eye of the other half was covered. The ‘before occlusion’ phase occurred after the ‘after occlusion’ phase because it was uncertain at the time of the experiment whether the nail varnish would have a long-term effect on the eye (subsequently we determined that there were no negative lasting effects, and the varnish was quickly removed by the stomatopods during routine grooming of the eyes with the maxillipeds).

To determine its optical density, the varnish was applied to a microscope slide using the same dabbing technique as used for application on the stomatopod eye. The transmission through slides both with and without the varnish was measured using a photospectrometer (USBQE6500, Ocean Optics Inc., Wesley Chapel, FL, USA) and analysed using Spectra Suite (Ocean Optics Inc.) and MATLAB version 2015b (The MathWorks). The varnish blocked 99.4% of light in the 420–700 nm range of the spectrum, which equates to an optical density of 2.2.

### Statistical analyses

All statistical analyses were conducted in R 3.0.25 (R Core Team, 2014). Because of non-normality, the median and 95% confidence interval (CI) of a distribution are quoted. Statistical analyses of gaze stabilization performance and saccadic response rate used a generalized linear mixed effects model (GLMM). Correlation between the rotation of the left and right eyes was calculated using Spearman's rank correlation coefficient and statistically analysed using a Wilcoxon signed-rank test, as was the saccadic response time.

## RESULTS

### Correlation between eyes during optokinesis

**Fig. 3.**
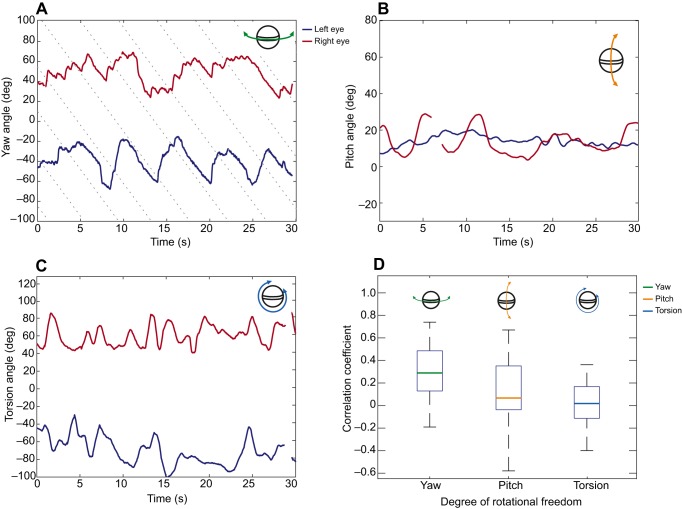
**Rotational responses of the left and right eyes to the horizontal motion of the visual field.** (A) The yaw rotation of the left (blue) and right (red) eyes of an individual during rotation of the drum showing stereotypical yaw optokinesis. The dotted lines indicate the progress of points on the surface of the drum, rotating in the yaw plane, but these lines do not necessarily represent specific stripe boundaries. (B) The pitch rotation and (C) torsion rotation of the same eyes during the same stimulus presentation. Note that the drum has no component of rotation in either pitch or torsion. (D) Boxplots displaying the range of Spearman's rank correlation coefficients between the left and right eyes of *O. scyllarus* in the yaw (green), pitch (yellow) and torsion (blue) degrees of rotational freedom (*n*=6).

During the ‘before occlusion’ phase, *O. scyllarus* performed stereotypical yaw optokinesis, as demonstrated in Fig. 3A. The median relative velocity ratio during the slow phase of optokinesis (when the eye rotated with the direction of the drum) across both eyes was *S*_y_=0.87±0.18 (median±95% CI, *n*=6), demonstrating good gaze stabilization performance. There was no significant difference between the gaze stabilization performance of the left and right eyes (left: *S*_yb_=0.93±0.20, right: *S*_yb_=0.81±0.16, where subscript b indicates ‘before treatment’, GLMM, *n*=6, χ^2^=1.74, *P*=0.187). The eyes also showed pitch (Fig. 3B) and torsion (Fig. 3C) rotation in response to the rotating drum, despite its motion being purely in the horizontal direction associated with the yaw degree of rotational freedom of the eye. The yaw rotation of the left and right eyes during optokinesis were weakly correlated (Fig. 3D), with the median Spearman's rank correlation coefficient (*r*_y_) significantly different from 0 (*r*_y_=0.28±0.16 median±95% CI, Wilcoxon signed-rank test, *n*=6, *V*=21, *P*=0.031). The pitch and torsion rotations of the two eyes were not correlated; the median Spearman's rank correlation coefficient was not significantly different from 0 for either pitch (*r*_p_=0.06±0.21 median±95% CI, Wilcoxon signed-rank test, *n*=6, *V*=14 *P*=0.106) or torsion (*r*_t_=0.02±0.17 median±95% CI, Wilcoxon signed-rank test, *n*=6, *V*=15, *P*=0.438).

The weak but significant correlation between the yaw rotation of the left and right eyes during optokinesis is substantially stronger than would be expected from two completely independent individuals viewing the same stimulus. For comparison, the Spearman rank correlation coefficient was calculated for the yaw rotations of the eyes of all possible combinations of different individuals (*r*_yy_). The median Spearman rank correlation coefficient for this independent correlation was *r*_yy_=0.07±0.06 (median±95% CI, *n*=6), indicating no correlation.

### Effect of covering an eye on the optokinetic response

**Fig. 4.**
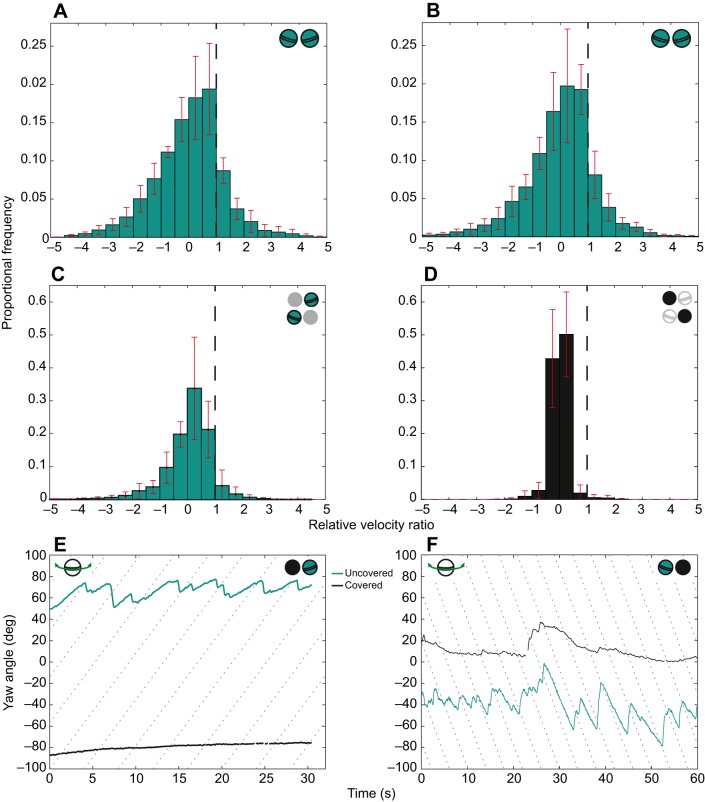
**The effect of covering one eye on the gaze stabilization response.** (A) The average distribution of the relative velocity ratio during both the slow and fast phases of yaw optokinesis for the eye from all six individuals in the ‘uncovered’ group during all trials (clockwise and anticlockwise) before occlusion. (B) The average distribution of the relative velocity ratio during both the slow and fast phases of yaw optokinesis for the eye from all six individuals in the ‘covered’ group before occlusion. Note that before occlusion, neither eye is occluded but they are grouped according to their treatment after occlusion. (C,D) The average distribution of the relative velocity ratio for the eyes of all six individuals in (C) the ‘uncovered’ and (D) the ‘covered’ groups after occlusion (i.e. as for A and B, respectively, but after occlusion). ‘Perfect’ gaze stabilization (relative velocity ratio=1) is indicated by the dashed vertical line. A–D show data from six repeated trials from a single eye from six individuals (*n*=6); red error bars are the standard deviation of the data in each abscissa interval (width 0.5). (E) When one eye is covered (in this case, the left eye), the contralateral eye (cyan line) continues to perform yaw optokinesis, while the covered eye (black line) remains stationary. (F) Alternatively, the covered eye (the right eye in this case; black) performs a yaw rotation that does not fit the optokinetic profile.

Before occlusion there was no significant difference in the yaw gaze stabilization performance between the two eyes (covered before: *S*_ycb_=0.89±0.18, uncovered before: *S*_yub_=0.83±0.18, GLMM, *n*=6, χ^2^=0.06, *P*=0.804; Fig. 4A,B). Note that rather than each of the eyes of the six individuals in these analyses being grouped as ‘left’ or ‘right’, they are grouped as ‘uncovered’ or ‘covered’ according to their treatment after occlusion, even though before occlusion, both eyes are uncovered. Covering an eye caused major changes in its movement, as shown by the substantial difference in the distribution of the relative velocity ratios in the covered (*S*_yca_) and uncovered (*S*_yua_) eyes (covered after: *S*_yca_=0.13±0.07 median±95% CI, uncovered after: *S*_yua_=0.44±0.19 median±95% CI; Fig. 4C,D) after occlusion. When one eye is completely covered, the uncovered eye continues to perform stereotypical yaw optokinesis, while the covered eye either remains stationary (Fig. 4E) or performs movements that do not fit the slow and fast phase criteria for optokinetic nystagmus (Fig. 4F).

**Fig. 5.**
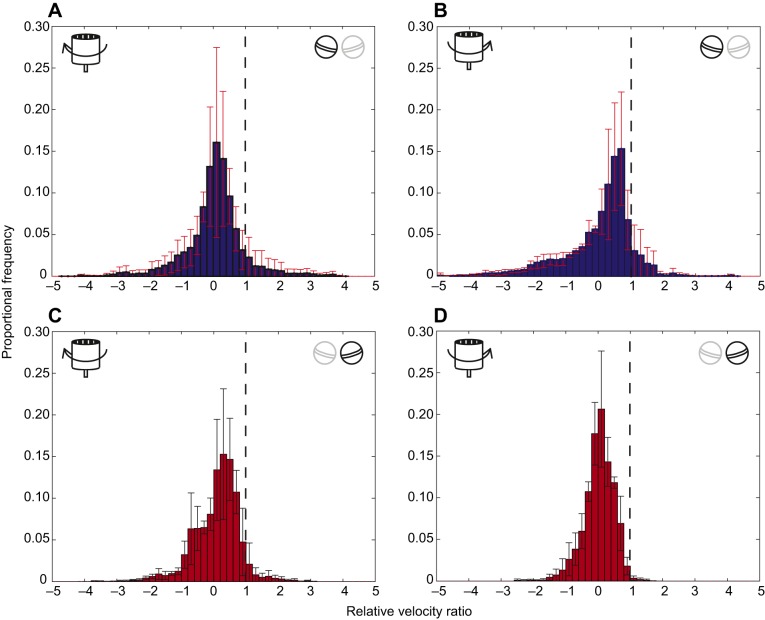
**The average distribution of the relative velocity ratio of the uncovered eye whilst the contralateral eye is occluded shows lateralization.** After occlusion, three individuals had their right eye covered and three had their left eye covered. All six animals were shown six presentations of the drum: three clockwise trials and three anticlockwise. The average distribution of the relative velocity ratio of the left eyes of all three individuals in the ‘uncovered’ group after occlusion differs significantly between (A) clockwise and (B) anticlockwise rotation of the drum. Similar results were obtained for the three right eyes in the ‘uncovered’ group after occlusion during (C) clockwise and (D) anticlockwise drum rotation. A–D show data from three repeated trials for a single eye from three individuals (*n*=3); red error bars are the standard deviation of the data in each abscissa interval (width 0.2).

Covering an eye had a significant negative effect on the uncovered eye. The gaze stabilization performance of the uncovered eye before occlusion (*S*_yub_) was significantly greater than that after occlusion (*S*_yua_) (uncovered before: *S*_yub_=0.83±0.18, uncovered after: *S*_yua_=0.44±0.19 median±95% CI, GLMM, *n*=6, χ^2^=44.36, *P*=0.001). Additionally, when both eyes were uncovered, the direction of drum rotation did not have a differential effect on the gaze stabilization performance of the left eye compared with the right eye [GLMM, interaction term between eye (left or right) and direction (clockwise or anticlockwise), *n*=6, χ^2^=0.17, *P*=0.676]. However, when one eye was covered, the direction of the drum did have a significant effect on the gaze stabilization performance of the uncovered eye, with the effect depending on which eye, left or right, was uncovered (GLMM, interaction term between eye and direction, *n*=6, χ^2^=6.62, *P*=0.013). With the right eye covered, the left eye performed better gaze stabilization when the drum rotated anticlockwise than when it rotated clockwise (anticlockwise: 0.55±0.01, clockwise: 0.37±0.02 median±95% CI). Conversely, with the left eye covered, the right eye performed better duringclockwise trials than during anticlockwise trials (clockwise: 0.49±0.01, anticlockwise: 0.34±0.01 median±95% CI). This asymmetry is clear in the distributions of the relative velocity ratios for the left and right eyes during clockwise and anticlockwise trials (Fig. 5).

### Effect of covering an eye on the response to a looming stimulus

**Fig. 6.**
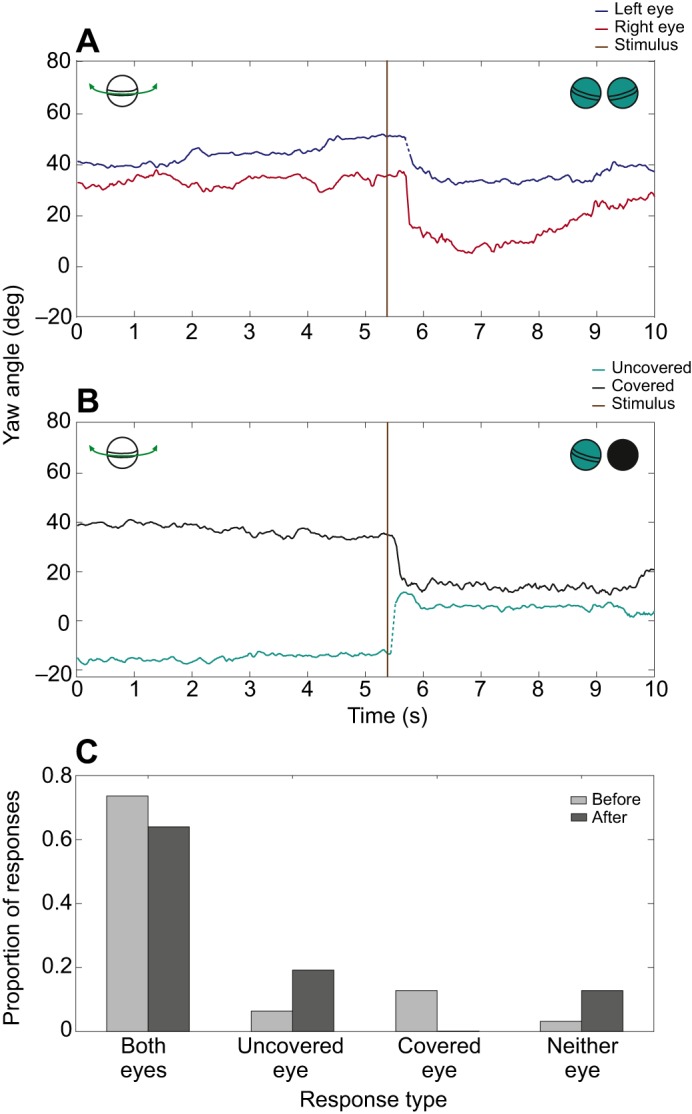
**The effect of covering one eye on the response to a looming stimulus.** (A) An example of a startle saccade in the yaw degree of rotational freedom involving both the left (blue line) and right (red line) eyes in response to a high-contrast looming stimulus (onset at brown line) when both eyes were uncovered. (B) A startle saccade to the same looming stimulus involving both eyes when the right eye (black line) was completely occluded and the left eye (cyan line) was uncovered. (C) The proportion of responses across all six animals before (light grey) and after (dark grey) occlusion involving both eyes, just the uncovered, just the covered or neither eye. As previously, though both eyes were uncovered before occlusion, the eyes are grouped by their treatment after occlusion (*n*=6).

*Odontodactylus scyllarus* performed startle saccades when presented with a black unpolarized (degree of polarization 0.006) looming stimulus on the white background of a CRT monitor both before and after eye occlusion. These saccades were performed with either one or both eyes, even after occlusion. No saccades were shown in response to the control stimuli. Fig. 6 shows examples of startle saccades involving yaw movements of both eyes in response to the high-contrast looming stimulus before occlusion, when both eyes were uncovered (Fig. 6A) and after occlusion, when (in this particular example) the right eye was covered (Fig. 6B). There was no significant difference in the response rate (a response is defined as either one or both eyes performing a startle saccade) before and after occlusion (before: 97%, after: 87%, GLMM, *n*=6, χ^2^=3.72, *P*=0.054). The eyes in the covered group responded in 90% of trials before occlusion, with the response rate falling to 67% after occlusion. There were no trials in which only the covered eye responded. Fig. 6C gives a graphical breakdown of the proportion of responses before and after occlusion in which the animals responded with both eyes, just the uncovered, just the covered or neither eye. The eye, left or right, had no significant effect on the response rate (GLMM, *n*=6, χ^2^=2.21, *P*=0.138).

There was no significant difference in the response times (defined as the time taken for an eye to begin a startle saccade after presentation of the looming stimulus) between the eyes before occlusion (difference in response time −0.34±4.82 s median±95% CI, Wilcoxon sign-ranked test, *n*=6, *V*=7, *P*=0.525). However, after one eye was occluded, the covered eye did respond significantly slower than the uncovered eye (difference in response time 4.88±17.21 s median±95% CI, Wilcoxon sign-ranked test, *n*=6, *V*=21, *P*=0.031).

## DISCUSSION

We have shown that the degree of independence between the two eyes of *O. scyllarus* depends on the visual task. The eyes act with a higher degree of independence during yaw optokinesis than during a startle saccade. Whilst an uncovered eye will drive a covered eye in a startle saccade in response to a high-contrast looming stimulus, an uncovered eye will not drive a covered eye during yaw optokinesis.

Although the uncovered eye does not drive the covered eye during yaw gaze stabilization, the two eyes of *O. scyllarus* do show weak but significant correlation when both eyes are uncovered. The median Spearman's rank correlation coefficient between the yaw rotations of the left and right eyes of the same animal was significantly higher than would be expected if the eyes of independent animals were compared during the same stimulus type. The weak correlation between eyes is perhaps unsurprising given the wide range of profiles of optokinetic nystagmus, in which both frequency and duration can vary substantially, even within an animal (see Figs 3A, 4E,F for comparison). While it was beyond the scope of this investigation to determine whether the correlation between the eyes was due to an active or passive interaction, there are several potential explanations. It is possible that the higher than expected degree of correlation stems from a mechanical interaction between the two eyes. Both eyes are connected to the rostral aspect of the head, which, as noted by Jones (1994), has some, albeit limited, range of yaw movement relative to the rest of the head. In addition, the joint at the base of a stomatopod eye stalk lacks condyles, and consists of a sheet of flexible arthroidial membrane from which the eyestalks are attached and suspended by muscles (Jones, 1994). As a result, the movements of one eye, especially towards the upper limit of its yaw range (>90 deg), may affect the position of the contralateral eye simply as a result of mechanical coupling.

The direction of drum rotation had an asymmetrical effect on the yaw gaze stabilization performance of an eye when its pair was covered, but not when both eyes were uncovered. This lateral asymmetry indicates that stomatopods may, at least partially, integrate visual information from the two eyes during yaw optokinesis. It has been shown previously that the two eyes of the blowfly use binocular input to enhance wide-field optic flow relating to specific types of self-motion (Krapp et al., 2001), so perhaps the visual system of stomatopods does something similar. Further to this is our finding in *O. scyllarus* that, when the left eye is covered, the right eye does not stabilize its gaze as well in response to a field of view moving from back to front (anticlockwise; temporo-nasal) as it does when the field is moving from front to back (clockwise; naso-temporal). Similarly, but in mirror image to the right eye, the left eye performs better gaze stabilization when the drum is rotating anticlockwise (front to back; naso-temporal) than it does when the drum rotates clockwise (back to front; temporo-nasal). This finding is converse to the trend shown by animals such as rabbits (Rademaker and Ter Braak, 1948) and guinea-pigs (Tauber and Atkin, 1968), which have afoveate, yoked eyes. In these animals, the optokinetic response to a field of view moving from front to back (naso-temporal) was suppressed or abolished when one of the eyes was covered. It is thought that this is a strategy that allows for retinal slip during translation of the body (i.e. locomotion), which gives rise to optic flow (Nakayama, 1985; Fritsches and Marshall, 2002). Optic flow is used by animals to determine self-motion during locomotion (Nakayama, 1985; Land, 1999b; Krapp et al., 2001; Fritsches and Marshall, 2002)*. Odontodactylus scyllarus* do have an acute zone (Horridge, 1978; Cronin, 1986; Marshall and Land, 1993), the compound eye equivalent of a fovea (Land and Eckert, 1985; Land, 2014; Marshall et al., 2014), which may explain why there is no reduction in gaze stabilization performance when the drum rotates naso-temporally, but this does not explain the reduction in performance during temporo-nasal drum rotation. The animals tested in this work were stationary; little is known about stomatopod vision during locomotion and, as yet, the role of optic flow in determining self-motion is unclear.

In response to a looming stimulus, both eyes typically perform a startle saccade. Even when one eye is covered, the uncovered eye will drive the covered eye in a startle response. There are several possible explanations for the apparent need to align two eyes as a consequence of a startle response. By using two eyes rather than one, the animal may have more chance of correctly analysing a potential threat such as a looming predator and therefore have a greater chance of escape. Unusually, in order to obtain spectral and polarization information from a scene, stomatopods perform scans in which the eye is drawn over the visual scene (Land et al., 1990) to expose different categories of midband photoreceptors to elements of the visual scene in a serial fashion. It is not clear whether vision is supressed in the hemisphere regions of the eye during these scans, but by using two eyes, the stomatopod may be reducing the time it takes to obtain all polarization and spectral information from a scene, and possibly reducing the time other visual functions are suppressed. Additionally, it is likely that there is an optimum scanning direction that depends on the spectral and spatial–temporal features of a scene. After the initial saccade, in which the eyes are yoked, the ocular independence in the torsional degree of rotation freedom may increase the likelihood that at least one of the eyes is already in the pose for scanning in the optimum direction, and thus can quickly obtain the maximum amount of information from a scene.

Alternatively, the pairing of the two eyes at the end of a startle response and their movement into a stereotypical pose, in which the eyes are aligned, may aid the stomatopod when judging distances. Stomatopods of the smasher variety, such as *O. scyllarus*, use strikes from their club-like raptorial appendages for both food and defence (Caldwell and Dingle, 1976). When assessing a possible food source, or a potential threat from a conspecific rival, it is crucial for the stomatopod to be able to quickly and accurately determine a salient object's distance from itself in order to maximize the impact of its strike. There is some evidence that stomatopods are able to judge distances with a single eye (Schiff et al., 1985, 1986; Schiff, 1996) but there is limited information about how this is achieved and, to date, neither binocular nor monocular stereopsis has been conclusively shown in stomatopods. Nevertheless, as there is extensive overlap in the fields of view between the left and right eyes and the hemispheres within an eye, stereopsis remains a possibility. The error in binocular depth perception is proportional to the interocular distance and the tangent of the interommatidial angle (Wilcox and Harris, 2010). If the dorsal and ventral hemispheres are treated effectively as two eyes, the interocular distance is roughly four times smaller than that between the left and right eyes after a startle saccade (Marshall and Land, 1993). Following the logic of Wilcox and Harris (2010), when a stomatopod views an object at a distance of 50 mm, the approximate error in that object's distance from the animal when viewed monocularly is ca. ±33 mm, whereas the error when both eyes are used falls to just ca. ±6 mm. These numbers are approximate, but they demonstrate the benefit a stomatopod may gain from binocular stereopsis over monocular stereopsis mediated by simultaneous viewing of a scene by the two hemispheres of one eye.

Several species of animals show pronounced visual field lateralization, in which one eye is preferred, or shows a significantly higher performance than the other during a specific task. For instance, chicks prefer to use their left eye to target a food source when it is clearly visible, but use the right eye when the food source is obscured (Tommasi and Andrew, 2002), while honeybees predominantly use their right eye when learning the association between a colour stimulus and a food reward (Letzkus et al., 2008). The octopus *Octopus vulgaris* also shows lateralized eye use, but while individuals show a preference for using either the left or right eyes, there is no predominant handedness at the population level (Byrne et al., 2004). The asymmetry in visual behaviours is suggestive of a lateralization of brain function (Bradshaw and Nettleton, 1981; Land, 2014). Although stomatopods show some lateral asymmetry between the two eyes during gaze stabilization in response to clockwise and anticlockwise rotation of the drum when one eye is covered, we did not find a substantial lateralization effect; when uncovered, one eye did not perform significantly better than the other during gaze stabilization or have a higher response rate to the looming stimulus. It is possible that, like the octopus, individual *O. scyllarus* show a visual handedness, but across the examined sample of animals there was no significant visual bias. A further study incorporating a greater number of individuals is required for a more in-depth investigation into possible lateralization of the stomatopod visual field, particularly if the handedness of individual animals is to be investigated.

This investigation has taken a behavioural psychophysical approach to investigate the extent of the independence between the two stomatopod eyes. The findings have raised several questions that now require a neurophysiological approach to be resolved. The discovery that a covered eye is not driven by an uncovered eye during yaw optokinesis would suggest that computation of motion vision in a single stomatopod eye occurs in its local peripheral visual system, possibly in one of the three optic lobes in the eye stalk (Kleinlogel and Marshall, 2005; Marshall et al., 2007). In contrast, the finding that an uncovered eye will drive a covered eye in a startle saccade perhaps suggests that the neural networks responsible for detecting looming motion integrate visual information from the two eyes, as well as co-ordinating a binocular response. As a startle response may lead to an escape reflex, it is very likely that such a response is processed more centrally in the visual system.

### Conclusions

The extent to which stomatopod eyes are independent of one another depends on the visual task. Different types of response are likely to follow different neural pathways, which are revealed by the disparate levels of independence between the two eyes. Such a strategy involving a hierarchy of interaction between two eyes may have implications for the optimal performance of autonomous multi-camera systems, such as those commonly deployed on robotic platforms.
